# Cross-Sectional Association between Estimated Hardness of the Habitual Diet and Depressive Symptoms in Older Japanese Men

**DOI:** 10.3390/nu15133034

**Published:** 2023-07-04

**Authors:** Aya Fujiwara, Ami Fukunaga, Kentaro Murakami, Yosuke Inoue, Tohru Nakagawa, Shuichiro Yamamoto, Maki Konishi, Tetsuya Mizoue

**Affiliations:** 1 Department of Epidemiology and Prevention, Center for Clinical Sciences, National Center for Global Health and Medicine, 1-21-1 Toyama, Shinjuku-ku, Tokyo 162-8655, Japan; 2 Department of Nutritional Epidemiology and Shokuiku, National Institutes of Biomedical Innovation, Health and Nutrition, 3-17 Senriokashinmachi, Settsu-shi 566-0002, Osaka, Japan; 3 Department of Public Health and Health Policy, Graduate School of Biomedical and Health Sciences, Hiroshima University, 1-2-3 Kasumi, Minami-ku, Hiroshima 734-8553, Japan; 4 Department of Nutritional Epidemiology and Behavioural Nutrition, Graduate School of Medicine, The University of Tokyo, 7-3-1 Hongo, Bunkyo-ku, Tokyo 113-0033, Japan; 5 Hitachi Health Care Center, Hitachi, Ltd., 4-3-16 Osecho, Hitachi-shi 317-0076, Ibaraki, Japan

**Keywords:** dietary hardness, mastication, chewing, depressive disorder, Japan

## Abstract

This cross-sectional study aimed to investigate the association between dietary hardness and depressive symptoms in older Japanese men. Participants were 1487 men aged 60–69 years enrolled in the baseline survey of the Hitachi Health Study II (2017–2020). Habitual dietary intake was estimated by a brief-type, self-administered diet history questionnaire. Dietary hardness was defined as the magnitude of masticatory muscle activity necessary to consume solid foods. The participants who scored ≥ 9 points on a short version of the Center for Epidemiologic Studies Depression Scale were considered to have depressive symptoms. The prevalence of depressive symptoms was 12.7%. The ORs (95% CIs) for depressive symptoms in the third tertile of dietary hardness were significantly lower after adjustment for sociodemographic and lifestyle-related variables and mood-modulating nutrients (ORs [95% CIs]: 0.93 [0.63, 1.36] and 0.58 [0.35, 0.97] for the second and third tertile, respectively [*p*-value for trend = 0.04]). Dietary hardness was inversely associated with the prevalence of depressive symptoms in older Japanese men. Future studies should confirm these findings and clarify the role of consuming a hard diet in preventing depressive disorders.

## 1. Introduction

The importance of late-life health for older generations is expected to increase as the global population ages, especially in high-income countries. The prevalence of individuals aged ≥ 60 years is projected to increase from 24% (301 million) in 2019 to 34% (439 million) in 2050 [1]. There are multiple risk factors for mental disorders at any point in life. However, older people may experience more major risk factors for depressive disorders than their younger counterparts, such as retirement, social isolation, bereavement, poor health status, and disability [2,3] In high-income countries, 4.4% (11 million) of individuals aged 60–89 years had depressive disorders in 2019. These disorders accounted for 1.1% of disability-adjusted life years and 4.4% of years lived with a disability [4]. Japan has the highest prevalence of individuals aged > 60 years in the world (35% in 2019) [1]. Given the substantial impact of depressive disorders on population health in developed countries where the population is rapidly ageing, identifying modifiable factors of depressive disorders in these countries is essential.

Mastication, i.e., voluntary rhythmic movements of the lower jaw by the masticatory muscles, has been suggested to improve depressive symptoms, as well as other physical activities [5]. Experimental studies have reported that participants (e.g., healthy volunteers or university students) with gum chewing intervention had lower levels of depressive symptoms than controls with no intervention in the UK [6,7], Japan [8], and Turkey [9]. Among healthy workers in the UK, Smith et al. [10] reported a lower prevalence of depressive symptoms in frequent gum chewers in a dose–response manner. These data raise the possibility that masticatory activity may prevent the onset and development of depressive disorders and motivate us to investigate the influence of a hard diet, which increases masticatory movement, on mental health status.

Dietary hardness can be used as a proxy for masticatory muscle activity required for habitual diet consumption. Comprehending the association between hardness of the habitual diet and depressive symptoms could provide valuable insights into preventing depressive disorders. As far as we know, no study has addressed the influence of dietary hardness on depressive symptoms. Murakami et al. [11] developed an algorithm to estimate dietary hardness using a self-administered diet history questionnaire (DHQ), and Okubo et al. [12] modified the algorithm for the use of the brief-type of DHQ (BDHQ). They examined the association with weight status [11], premenstrual symptoms [13], and cognitive functions [12]. The present cross-sectional study aimed to investigate the association between hardness of the habitual diet and depressive symptoms in older Japanese men. The hypothesis was that higher dietary hardness would be associated with a lower prevalence of depressive symptoms.

## 2. Materials and Methods

### 2.1. Study Design and Participants

The data for this cross-sectional study were obtained from a baseline survey of the Hitachi Health Study II, an ongoing prospective cohort. The Hitachi Health Study II mainly aimed to examine the risk factors for dementia and other non-communicable diseases among current and former employees and their spouses at Hitachi, Ltd. in Ibaraki prefecture. We invited individuals aged ≥ 60 years (as of 31st March each year) who had a health check-up, including cognitive function screening by the company to participate in the baseline survey.

**Figure 1:**
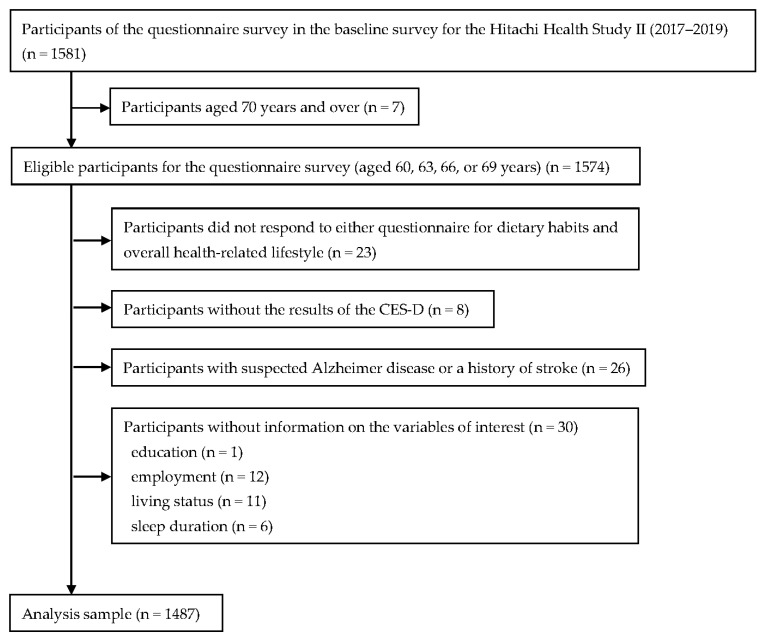
Flowchart of participants included in the present analysis: Hitachi Health Study II.

The baseline survey was conducted from April 2017 to March 2020. As part of the health check-up, anthropometric and biochemical measurements were performed, and participants’ medical history information was collected. Participants aged 60, 63, 66, or 69 years were further given two questionnaires to complete, which asked about their dietary habits and overall health-related lifestyle. The target population in the present analysis included individuals who completed the questionnaire (n = 1581). Due to the low prevalence of women in the original sample (10.4%), no female participants were included in the present study. For analysis, we excluded those aged ≥ 70 years (n = 7) and those without the results of either the questionnaire survey (n = 23) or measurement of depressive symptoms (n = 8). We also excluded those with suspected Alzheimer’s disease (mentioned below) or a medical history of stroke (n = 26). This is because the former may have difficulties providing accurate responses to questionnaires, while the latter may experience impaired masticatory ability due to stroke. After excluding those without information on the variable of interest (n = 30), the final analysis sample comprised 1487 men (Figure 1).

This study was conducted in adherence to the guidelines laid down in the Declaration of Helsinki. Ethical approval for all procedures involving human participants was obtained from the Ethics Committee of the National Center for Global Health and Medicine (approval number: NCGM-G-002208) and Hitachi Health Care Center. Written informed consent was obtained from all participants prior to participation.

### 2.2. Estimation of Dietary Hardness

Information on habitual dietary intake was obtained from the participant’s responses to a brief-type, self-administered diet history questionnaire (BDHQ) [14,15]. The BDHQ was designed to estimate the daily intake of commonly consumed 58 food items in Japan, including 38 solid foods, 12 beverages, and 8 seasonings [14]. The fixed-portion size questionnaire enquires about the frequency of selected food intake over the past month. To calculate energy and nutrient intake, an ad hoc computer algorithm specific to the BDHQ was employed [15], using the Standard Tables of Food Composition in Japan, 2010 [16]. Previous studies assessing the relative validity have demonstrated satisfactory rankings of individuals based on their energy-adjusted intake of various food groups and nutrients by the density method [14,15]. Briefly, the median (interquartile range) of Spearman’s correlation coefficient between the BDHQ and 16-day dietary records was 0.48 (0.33–0.56) for food groups among Japanese men aged 32–76 years [14], while the median (interquartile range) of Pearson’s correlation coefficient for nutrients was 0.56 (0.41–0.63) [15].

Dietary hardness, which reflects the masticatory muscle activity required for consuming a habitual diet, was defined following established approaches in previous studies [11,12,17,18]. To calculate the hardness of each participant’s habitual dietary intake (mV·s/day), we multiplied the hardness of each food item in the BDHQ (mV·s/cm^3^) by its volume consumed (cm^3^/day) and then summed the products [11,12]. Of 38 solid food items in the BDHQ, 34 were directly matched to an equivalent food item with information on masticatory muscle activity (mV·s/2.197 cm^3^) [18]. For the remaining 4 items, the values from similar food items were used as proxies [11,12]. Subsequently, each value was divided by 2.197 to obtain the hardness of each food item (mV·s/cm^3^) [11,12]. Considering the significant impact of cooking methods on the hardness of vegetables [18], we incorporated the observed ratio of consumption between raw and cooked forms (unpublished observations by S. Sasaki, 2006) as much as possible during the matching procedure [11,12]. Food volume consumed (cm^3^/day) was estimated using the weight in grams (g/day) obtained from the BDHQ. For this estimation, we assumed a uniform density of 1 (g/cm^3^) for all foods [11,12].

Given the variations in dietary intake attributable to different body sizes and energy requirements [19] and to mitigate the impact of misreporting [20], as well as the high correlation observed between energy intake and the crude estimate of dietary hardness in this population (Pearson correlation coefficient: r = 0.87), we energy-adjusted both dietary hardness and dietary intake by density methods [21]. Although in previous studies, dietary hardness was adjusted for total energy intake [11,12], energy-adjusted dietary hardness was shown as the value per 4184 kJ of energy intake from solid foods and seasonings due to the non-contribution of beverages to dietary hardness estimation. Meanwhile, energy-adjusted dietary intake was provided as units/4184 kJ of total energy intake.

### 2.3. Assessment of Depressive Symptoms

The short version of the Center for Epidemiologic Studies Depression Scale (CES-D 11) [22,23] assessed depressive symptoms. This measure consists of 11 items from the original 20-item version, which asks individuals about their experience of symptoms commonly associated with depression within the preceding week. The items used in this study were derived from the Japanese 20-item version [24]. Consistent with a previous study [25] which employed arithmetic conversion to establish a cut-off score, participants scoring ≥ 9 points were categorised as having depressive symptoms.

### 2.4. Assessment of Covariates

The health check-up data, including medical history, were referred to collect information on anthropometric measurements, lifestyle-related variables, and history of the disease. Body height (in 0.1 cm) and weight (in 0.1 kg) were measured while the participants were in light clothes and without shoes. Body mass index (BMI, kg/m^2^) was calculated by dividing body weight by the square of height. Fasting plasma glucose levels were determined using the glucose oxidase enzyme electrode method (A&T, Tokyo, Japan), while haemoglobin A1c (HbA1c) levels were measured using high-performance liquid chromatography (HLC723-G9, TOSOH, Tokyo, Japan). Diabetes was defined as meeting one or more of the following criteria: fasting plasma glucose (FPG) ≥ 126 mg/dL, HbA1c ≥ 6.5%, and self-reported use of medications for diabetes [26]. Lifestyle-related variables encompassed smoking status, alcohol consumption evaluated by the frequency and amount, and habitual exercise. Participants with a previous history of cancer, myocardial infarction, chronic hepatitis, or kidney disease were considered to have a chronic disease history.

Cognitive function was evaluated using a computerised test battery (MSP-1100, Nihon Kohden Corporation, Tokyo, Japan) designed to screen individuals who may be at risk of Alzheimer’s disease [27]. The MSP-1100 was derived from the revised version of Hasegawa’s Dementia Scale [28]. It comprised four tasks aimed at assessing different aspects of cognitive function in participants: temporal memory (three items), temporal orientation (four items), three-dimensional visual-spatial perception (two items), and short-term memory (three items) [27]. Each item was assigned a score of 1 (for the former three tasks) or 2 (for the latter one task) points for every correct response. The total score ranged from 0 to 15 points, with higher scores indicating better cognitive function. We defined participants scoring ≤ 13 points on the MSP-1100 during the initial test as having a cognitive dysfunction [27,29]. Additionally, according to the instructions of the MSP-1100, participants scoring ≤ 12 points were asked to undergo a secondary screening test (TDAS-1100; Nihon Kohden Corporation, Tokyo, Japan) [30]. The TDAS was established based on the ADAS-cog, a subscale of the Alzheimer’s Disease Assessment Scale [31]. It consisted of nine tasks for examining an ability in participants: word-recognition (twenty-four items × three trials), following a command (two trials), orientation (four items), visual-spatial perception (four items), naming finger (five items), object recognition (three items), accuracy of the order of a process (one trial), money calculation (three trials), and clock time recognition (three items) [30]. Each item was assigned a score of 1 point, except for accuracy of the order of a process (5 points), for every incorrect response. The total score ranged from 0 to 101 points, with a lower score indicating better cognitive function. We defined participants scoring ≥ 14 points on the TDAS-1100 as having suspected Alzheimer’s disease based on the instructions.

The overall health-related lifestyle questionnaire was used to obtain information on the following sociodemographic and lifestyle-related variables: age, education, current employment, living alone, and sleep duration.

### 2.5. Statistical Analysis

The statistical analyses for this study were conducted using SAS version 9.4 (SAS Institute Inc., Cary, NC, USA). Two-tailed *p*-values were reported for all analyses, and statistical significance was defined as *p* < 0.05. Descriptive data for continuous variables were presented as mean and standard deviation (SD), while categorical variables were presented as numbers and percentages of participants. Energy-adjusted dietary hardness (mV·s/4184 kJ) was divided into tertiles to compare the participants’ characteristics. To investigate the association between dietary hardness and dietary intake, we showed the mean and standard error (SE) for each dietary hardness category with adjustment for age.

Logistic regression analysis was performed to estimate the odd ratio (OR) and 95% confidence interval (CI) for depressive symptoms across each tertile of dietary hardness with the lowest category as the reference. In this study, three models were constructed. Model 1 was adjusted for age. Model 2 was further adjusted for the potential confounding factors, including BMI, education, current employment, living alone, smoking, alcohol consumption, sleep duration, habitual exercise, chronic disease history, diabetes, cognitive dysfunction [32,33], and energy intake [34]. Model 3 included additional adjustment for intake of *n*-3 polyunsaturated fatty acid (PUFA), vitamins B_6_, B_12_, folate, Mg, and Zn [35,36,37]. This adjustment aimed to assess whether the association observed between dietary hardness and depressive symptoms would be independent of these nutrient intakes. Linear trends were evaluated by assigning each participant a median value for each tertile as a continuous variable.

In the nature of cross-sectional analysis, it is possible for dietary intake, and thus its hardness, to be influenced by mental status, leading to reverse causality [38]. Therefore, we confirmed whether the results would be altered by excluding participants with a history of mental disorders (n = 32) in the sensitivity analysis.

## 3. Results

### 3.1. Associations between Dietary Hardness and Selected Characteristics

The participants had a mean (SD) age of 63.5 (3.5) years and a mean (SD) dietary hardness of 221 (29) mV·s/4184 kJ. The prevalence of depressive symptoms evaluated using the CES-D 11 was 12.7%. Participants with higher dietary hardness tended to be older, have a lower BMI, be unemployed, live with someone, be current smokers, consume alcohol, sleep for longer hours, exercise more habitually, and report diabetes. In contrast, they were less likely to have a chronic disease history (Table 1).

### 3.2. Associations between Dietary Hardness and Dietary Intake

Dietary hardness was not significantly associated with total energy intake (Table 2). Dietary hardness was positively associated with the intake of all nutrients investigated in this study. Dietary hardness was positively associated with the intake of potatoes, pulses, vegetables, fruits, fish and shellfish, meats, alcoholic beverages, and seasonings and negatively associated with the intake of bread, confectioneries, and sugar-sweetened beverages.

### 3.3. Associations between Dietary Hardness and the Prevalence of Depressive Symptoms

The association between dietary hardness and the prevalence of depressive symptoms is shown in Table 3. The ORs (95% CIs) for depressive symptoms in the third tertile of dietary hardness were significantly lower after adjustment for age: Model 1: 0.93 (0.65, 1.32) and 0.55 (0.37, 0.81) in the second and third tertile compared with the first tertile, respectively (*p*-value for trend = 0.003). This association remained significant after further adjustment for sociodemographic and lifestyle-related variables (Model 2: 0.92 [0.64, 1.33] and 0.58 [0.38, 0.87], *p*-value for trend = 0.01) and potentially protective nutrients against depressive symptoms (Model 3: 0.93 [0.63, 1.36] and 0.58 [0.35, 0.97], *p*-value for trend = 0.04). In the sensitivity analysis excluding participants with a history of mental disorders, the observed association did not change; ORs (95% CIs) in the second and third tertile compared with the first tertile were 0.93 (0.63, 1.37) and 0.56 (0.33, 0.95) (*p*-value for trend = 0.04) in Model 3.

## 4. Discussion

Among 1487 older Japanese men included in the Hitachi Health Study II, we observed that participants in the highest tertile of dietary hardness were less likely to have depressive symptoms than those in the lowest tertile. This association remained significant after adjusting for both sociodemographic and lifestyle-related variables and the dietary intake of potentially protective nutrients. As far as we know, this study first investigated the association between dietary hardness and depressive symptoms.

The inverse association between dietary hardness and depressive symptoms observed in our study is consistent with findings from interventional studies; an intervention of 7- to 19-day gum chewing had a favourable effect on depressive symptoms in healthy volunteers in the UK [6] and university students in the UK [7], Japan [8], and Turkey [9]. Our results also align with those of Smith et al. [10], who performed a cross-sectional study among 388 healthy workers in the UK. The authors reported an inverse association between the frequency of gum chewing and depressive symptoms (non vs. ≤1 time/week vs. ≥5 days/week: 23.4% vs. 16.4% vs. 10.0%, respectively; chi-squared *p*-value < 0.05). The present study on dietary hardness extends the favourable role of mastication in depressive mood suggested by gum chewing studies to diet in daily life.

In our study, dietary hardness was positively associated with the intake of mood-modulating nutrients (i.e., *n*-3 PUFA, VB6, VB12, folate, Mg, and Zn) (Table 2). This finding suggests that the association between dietary hardness and depressive symptoms may be attributed to the favourable nutrient profile of a hard diet rather than hardness itself. Nevertheless, the observed association between dietary hardness and depressive symptoms was virtually unchanged after adjusting for the intake of these nutrients (Table 3). This finding supports the hypothesis that dietary hardness, per se, is associated with depressive symptoms.

The mechanism underlying the association between dietary hardness and depressive symptoms remains largely unknown. However, the present findings may be explained by the favourable effects of enhanced masticatory activity. In a human experiment, gum chewing was shown to activate the ventral part of the prefrontal cortex and subsequently increase the blood levels of serotonin [39], a neurotransmitter responsible for regulating mood. In another human experiment [40], gum chewing also reduced salivary cortisol levels, anxiety, and psychological stress during a stress-loading intervention, suggesting that mastication can affect mood via the hippocampus and hypothalamus–pituitary–adrenal axis [41]. Meanwhile, animal experiments reported that compared to normal–diet feeding mice, soft–diet feeding mice reduced the mRNA expression [42] and protein level [43] of a brain-derived neurotrophic factor in the hippocampus, which is known to promote neuronal cell proliferation, differentiation, survival, and synapse formation [44]. These results suggested that increased masticatory activity while consuming a diet with greater hardness can improve mood and may be resilient to mental distress.

This study had several limitations to consider. First, dietary hardness was assessed from the solid food group intake estimated using the BDHQ. While the BDHQ has been validated for estimating the intake of selected foods and nutrients [14,15], no previous validation study has been conducted regarding estimates of dietary hardness derived from the BDHQ [18]. It might be problematic to assume the equivalence of masticatory muscle activity required for consuming specific food items between our older participants and the younger reference population, for whom a published database was developed [18]. Moreover, the BDHQ does not collect sufficient information on how people cook or digest each food item, which may have contributed to interindividual variations in estimated dietary hardness. Second, despite the adjustment for a wide range of potential confounding factors, the influence of unmeasured or residual confounding factors could not be ruled out. Third, the assessment of depressive symptoms was conducted using the Japanese version of the CES-D 11. This questionnaire has not been validated in a Japanese population, although we used items from the Japanese version of the 20-item CES-D [24], which has been validated. Fourth, the sample size was not calculated in advance of the present analysis because the original study was designed to investigate the associations of various exposures with dementia and other non-communicable diseases in a fixed population. Nevertheless, the present study has sufficient power to detect the association between hardness of the habitual diet and depressive symptoms with statistical significance. Fifth, we confirmed that excluding participants with a history of mental disorders (n = 32) did not alter the association. Therefore, the present findings are unlikely to be explained by individuals diagnosed with mental disorders and consumed a diet of lower hardness. However, this does not imply a lack of reverse causality because of the uncertain temporal order of exposure and outcome in the present cross-sectional setting. Finally, the present study enrolled participants who had a health check-up at a Japanese company and might not be representative of the general population of older men in Japan.

## 5. Conclusions

In conclusion, hardness of the habitual diet was inversely associated with the prevalence of depressive symptoms among older Japanese men, independent of potentially protective nutrient intake. As far as we know, this study first investigated the association between hardness of the habitual diet and depressive symptoms. Our findings suggested the possibility of beneficial effects of dietary hardness on mental health status. Further experimental and observational studies are necessary to clarify the causal mechanism for our findings, including a dose–response relationship between the consumption of a diet with a variety of hardness with mental health status and the role of consuming a hard diet in preventing mood disorders.

## Figures and Tables

**Table 1:** Selected characteristics of 1487 old Japanese men according to tertile of energy-adjusted dietary hardness: the Hitachi Health Study II.

	T1 (n = 495)		T2 (n = 496)		T3 (n = 496)	
	n	(%)	n	(%)	n	(%)
Dietary hardness (mV·s/4184 kJ), mean, SD *	192	13	219	6	253	22
Age (years), mean, SD	63.2	3.5	63.5	3.6	63.8	3.5
BMI (kg/m^2^), mean, SD	24.4	3.3	24.2	3.2	23.9	2.8
Education (years)						
<10	17	(3.4)	24	(4.8)	25	(5.0)
10 to 12	275	(55.6)	237	(47.8)	255	(51.4)
≥13	203	(41.0)	235	(47.4)	216	(43.5)
Current employment	357	(72.1)	355	(71.6)	349	(70.4)
Living alone	37	(7.5)	36	(7.3)	30	(6.0)
Smoking status						
Never	151	(30.5)	147	(29.6)	136	(27.4)
Former	239	(48.3)	244	(49.2)	242	(48.8)
Current	105	(21.2)	105	(21.2)	118	(23.8)
Alcohol consumption (g of ethanol/day)						
None	146	(29.5)	124	(25.0)	99	(20.0)
>0 to <46	316	(63.8)	327	(65.9)	335	(67.5)
≥46	33	(6.7)	45	(9.1)	62	(12.5)
Sleep duration (h/d)						
<6	51	(10.3)	44	(8.9)	37	(7.5)
6 to <7	145	(29.3)	152	(30.6)	125	(25.2)
≥7	299	(60.4)	300	(60.5)	334	(67.3)
Habitual exercise	216	(43.6)	223	(45.0)	287	(57.9)
History of chronic diseases ^†^	62	(12.5)	49	(9.9)	44	(8.9)
Diabetes ^‡^	104	(21.0)	94	(19.0)	128	(25.8)
Cognitive dysfunction ^§^	41	(8.3)	32	(6.5)	38	(7.7)

SD, standard deviation; T, tertile; * Presented as a value per 4184 kJ of energy intake from solid foods (i.e., foods and seasonings). ^†^ Having a history of cancer, myocardial infarction, chronic hepatitis, or kidney disease. ^‡^ Defined as present when participants with FPG ≥ 126 mg/dL, HbA1c ≥ 6.5%, and/or self-report being currently under medical treatment for diabetes. ^§^ Participants scoring ≤ 13 points on the MSP-1100 were classified as having a cognitive decline.

**Table 2:** Dietary intake according to tertile of energy-adjusted dietary hardness (in mV·s/4184 kJ) in 1487 old Japanese men: the Hitachi Health Study II.

	T1 (n = 495)		T2 (n = 496)		T3 (n = 496)		
	Mean	SE	Mean	SE	Mean	SE	P for Trend *
Dietary hardness (mV·s/4184 kJ)							
Median	195		219		246		
Energy intake (kJ/day)	8222	104	8231	104	8174	104	0.73
Nutrients ^†^							
*n*-3 PUFA (g/4184 kJ)	1.3	0.0	1.4	0.0	1.5	0.0	<0.001
Vitamin B_6_ (mg/4184 kJ)	0.56	0.01	0.64	0.01	0.76	0.01	<0.001
Vitamin B_12_ (μg/4184 kJ)	4.2	0.1	5.0	0.1	6.1	0.1	<0.001
Folate (μg/4184 kJ)	145	2	167	2	214	2	<0.001
Magnesium (mg/4184 kJ)	123	1	134	1	154	1	<0.001
Zinc (mg/4184 kJ)	4.1	0.0	4.3	0.0	4.6	0.0	<0.001
Food groups (g/4184 kJ) ^†^							
Rice	162.3	2.9	181.5	2.9	166.5	2.9	0.44
Bread	23.7	0.7	17.3	0.7	11.9	0.7	<0.001
Noodles	41.6	1.3	45.6	1.3	44.0	1.3	0.22
Potatoes	14.5	0.7	17.9	0.7	19.2	0.7	<0.001
Sugar and sweeteners	2.9	0.1	2.5	0.1	2.6	0.1	0.37
Pulses	31.8	1.0	35.1	1.0	41.4	1.0	<0001
Vegetables	83.2	2.4	110.1	2.3	170.0	2.4	<0.001
Fruits	31.4	1.4	36.5	1.4	43.5	1.4	<0.001
Fish and shellfish	32.8	1.0	40.8	1.0	52.1	1.0	<0.001
Meats	32.9	0.9	34.6	0.9	37.2	0.9	<0.001
Eggs	20.8	0.6	20.1	0.6	22.3	0.6	0.09
Dairy products	66.6	2.6	60.0	2.6	70.6	2.6	0.22
Fat and oil	9.4	0.2	9.1	0.2	9.0	0.2	0.15
Confectioneries	42.5	0.9	26.2	0.9	17.0	0.9	<0.001
Fruit and vegetable juices	24.9	2.1	23.9	2.1	27.2	2.1	0.41
Alcoholic beverages	85.8	5.4	101.3	5.4	117.5	5.4	<0.001
Unsweetened tea and coffee	286.9	8.3	298.5	8.3	302.7	8.3	0.18
Sugar-sweetened beverages	34.6	2.2	23.3	2.2	21.0	2.2	<0.001
Seasonings	10.8	0.2	11.7	0.2	11.8	0.2	<0.001

SE, standard error; T, tertile. * A linear regression model was performed with the median value in each category of energy-adjusted dietary hardness (in mV·s/4184 kJ) as a continuous variable with adjustment for age (years, continuous). ^†^ Presented as a value per 4184 kJ of total energy intake.

**Table 3:** ORs (95% CIs) for depressive symptoms according to tertile of energy-adjusted dietary hardness in 1487 old Japanese men: the Hitachi Health Study II.

	T1 (n = 495)		T2 (n = 496)			T3 (n = 496)			P for Trend *
Dietary hardness (mV·s/4184 kJ), median	195		219			246			
Depressive symptoms, n (%) ^†^	76	(15.4)	70	(14.1)		43	(8.7)		
Model 1 ^‡^	1	(reference)	0.93	(0.65	1.32)	0.55	(0.37	0.81)	0.003
Model 2 ^§^	1	(reference)	0.92	(0.64	1.33)	0.58	(0.38	0.87)	0.01
Model 3 **	1	(reference)	0.93	(0.63	1.36)	0.58	(0.35	0.97)	0.04

T, tertile. * Logistic regression model was performed with the median value in each category of energy-adjusted dietary hardness (in mV·s/4184 kJ) as a continuous variable. ^†^ Participants scoring ≥ 9 points on the CES-D 11 were classified as having depressive symptoms [25]. ^‡^ Adjusted for age (years, continuous). ^§^ Further adjusted for BMI (kg/m^2^, continuous), education (<10, 10–12, ≥13 years), current employment (yes or no), living alone (yes or no), smoking status (never, former, or current), alcohol consumption (none, >0 to <46, or ≥46 g of ethanol/day), habitual exercise (yes or no), history of chronic diseases (yes or no), diabetes (yes or no), cognitive dysfunction (yes or no), and energy intake (kJ/day, continuous). ** Further adjusted for energy-adjusted intake of *n*-3 PUFA, VB_6_, VB_12_, folate, Mg, and Zn (unit/4184 kJ, continuous).

## Data Availability

The data are not publicly available but are available upon reasonable request to the corresponding author (fujiwaraay-tky@umin.ac.jp).
